# Ginsenoside-Rg3 inhibits the proliferation and invasion of hepatoma carcinoma cells via regulating long non-coding RNA HOX antisense intergenic

**DOI:** 10.1080/21655979.2021.1932211

**Published:** 2021-06-15

**Authors:** Zhongjian Pu, Fei Ge, Yajun Wang, Ziyu Jiang, Shilin Zhu, Shukui Qin, Qijun Dai, Hua Liu, Haiqing Hua

**Affiliations:** a Graduate School, Nanjing University of Chinese Medicine, Nanjing, Jiangsu, China; b Department of Oncology, Haian Hospital of Traditional Chinese Medicine, Haian, Jiangsu, China; c Department of Gastroenterology, Haian Hospital of Traditional Chinese Medicine, Haian, Jiangsu, China; d Department of Oncology, Hospital of Integrated Traditional Chinese Medicine and Western Medicine Affiliated to Nanjing University of Chinese Medicine, Nanjing, Jiangsu, China; e Department of Oncology, Bayi Hospital Affiliated to Nanjing University of Chinese Medicine, Nanjing, Jiangsu, China; f Department of Neurology, Haian Hospital of Traditional Chinese Medicine, Haian, Jiangsu, China; g Department of Orthopedics, Haian Hospital of Traditional Chinese Medicine, Haian, Jiangsu, China

**Keywords:** Ginsenoside-Rg3, lncRNA HOTAIR, hepatoma carcinoma, PI3K/AKT

## Abstract

Ginsenoside Rg3, a natural compound, has been reported to function as an anticancer agent for hepatoma carcinoma, while the mechanisms underlying the anticancer effects are not clear. Therefore, the objective of our study was to explore the impact of RG3 on cell migration and invasion by regulating the lncRNA HOX antisense intergenic (HOTAIR) expression involving PI3K/AKT signaling pathway. qRT-PCR was utilized to measure the mRNA expression of HOTAIR. Furthermore, HOTAIR overexpression plasmids were transfected to SMMC-7721 and SK-Hep-1 cells. Additionally, MTT assay was used to evaluate the proliferation of transfected cells. The scratch and transwell assays were used to determine the migration and invasion ability of the cell. The protein levels were determined with Western blot. lncRNA HOTAIR was overexpressed in SMMC-7721 and SK-Hep-1 cells. Ginsenoside-Rg3 reduced the level of lncRNA HOTAIR. Overexpressed lncRNA HOTAIR offset ginsenoside-Rg3 inhibited proliferation, migration and invasion of HCC cells. Furthermore, ginsenoside-Rg3 decreased the expression of p-AKT, p-PI3K, matrix metalloproteinase-2 (MMP2) and matrix metalloproteinase-9 (MMP9), which was reversed after the treatment of HOTAIR. LncRNA HOTAIR was overexpressed in SMMC-7721 cells. Ginsenoside-Rg3 could reduce the expression of lncRNA HOTAIR, resulting in the inhibited cell proliferation, migration and invasion. Furthermore, ginsenoside-Rg3 inhibited cell proliferation and invasion ability through the PI3k/AKT pathway. Thus, ginsenoside-Rg3 might be a potential and effective treatment for HCC.

## Graphical Abstract

**Figure:**
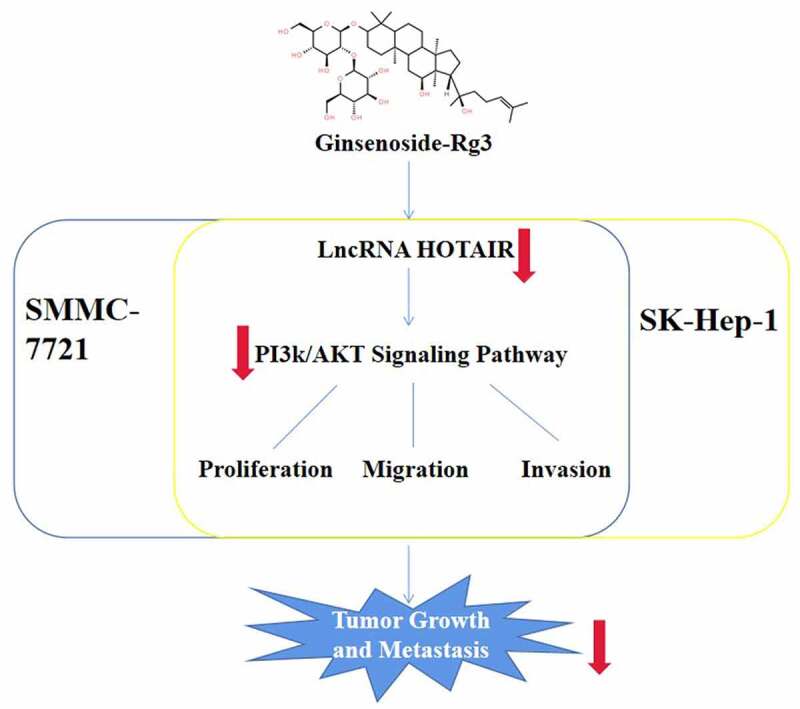

## Introduction

Hepatocellular carcinoma (HCC) is one of the most common cancers around the world, which has high incidence rate and mortality. Nearly 748,300 new cases and 695,900 deaths are reported per year worldwide [1]. Patients with a history of cirrhosis are most likely to develop to HCC [2]. The prevailing therapies for HCC are to rely on surgical treatment or local ablation [3]. However, the lack of specific symptoms in the early stage of HCC is one of the main reasons for the poor curative effect of HCC [4]. More than 60% of patients are diagnosed with advanced cancer with the status of metastasis and an overall 5-year survival rate of less than 16% [5]. Therefore, it is urgent to effectively retard the cell migration and invasion ability in order to prolong the survival rate and cure rate of patients with advanced HCC.

Long non-coding RNA (lncRNA), a single-stranded RNA containing more than 200 nucleotides, which lacks significant open reading frames, are unable to code for proteins [6]. Furthermore, lncRNAs are key regulators of cellular function through epigenetic regulation, miRNA sponging, enzyme cofactors and modulating of proteins [7]. Therefore, lncRNAs are a major class of RNA molecules which exert a powerful effect on various physiological and pathological processes involving in tumor growth, progression, and prognosis. In addition, lncRNAs could provide potential clues for developing novel therapeutic approaches for cancers [8]. Previous studies have found that lncRNA HOX antisense intergenic (HOTAIR) is significantly increased in cancerous tissues in comparison to normal tissues in gastric cancer, urothelial cell carcinoma and renal cell carcinoma [8–10]. Additionally, lncRNA HOTAIR is closely related to cell proliferation and migration ability [10]. In Di et al.’s study, lncRNA HOTAIR epigenetically inhibited the level of miR-122 in hepatocellular carcinoma via DNA methylation, which contributes to the activation of Cyclin G1 and promotes the malignant development of HCC [11]. Furthermore, the study determined that the high level of lncRNA HOTAIR was related to hepatocarcinogenesis and metastasis, and overexpression of lncRNA HOTAIR is also a predictor of tumor recurrence in HCC [12,13].

Ginsenoside Rg3 (C_42_H_72_O_13_; molecular weight 785.01) is an effective component extracted from ginseng [14]. Ginsenoside Rg3 is the first monomer of traditional Chinese medicine applied to the treatment of various kinds of tumors due to its tumor angiogenesis-inhibiting effect and interaction between endothelial cells and the extracellular matrix [15]. So far, ginsenoside-Rg3 has been regarded as an effective drug for postoperative recovery of most of the malignant tumors, which can significantly alleviate the recurrence and diffusion of tumors [16]. Ginsenoside-Rg3 not only suppresses the migration and invasion but also promotes the apoptosis of colorectal cancer cells through reducing the level of LncRNA CCAT1. Ginsenoside-Rg3 suppresses the migration and invasion of hepatoma cells through regulating ARHGAP9. Phosphoinositide-3-kinase (PI3K)/AKT signaling pathway is closely related to various factors, and its activation and inhibition regulate the important cell activity including cell proliferation, migration, invasion and so forth [17]. Furthermore, the cell migration and invasion-related genes are regulated by PI3K/AKT, such as metalloproteinase 2 (MMP2) and matrix metalloproteinase 9 (MMP9) [18]. However, the specific mechanism of ginsenoside-Rg3 on the lncRNA HOTAIR expression of HCC cells remains unclear.

Therefore, this study is the first to explore the effect and mechanisms of ginsenoside-Rg3 on lncRNA HOTAIR in hepatoma carcinoma cells. We hypothesized that ginsenoside-Rg3 inhibits the lncRNA HOTAIR expression, growth and metastasis of the HCC cells via regulating the PI3k/AKT signaling pathway.

## Materials & methods

### Cells

SMMC-7721, SK-Hep-1 and HEK293T cells were purchased from ATCC and cultured in RPMI-1640 medium containing 10% fetal bovine serum, 100 U/ml penicillin, and 100 μg/ml streptomycin to the 70–80% confluence for the following experiments at 37°C with 5% CO_2_.

### Transfection

SMMC-7721 and SK-Hep-1 cells were seeded in 24-well plates. Furthermore, there were four groups in the cell experiment: control group, ginsenoside-Rg3 groups, HOTAIR overexpression group, and ginsenoside-Rg3+ HOTAIR control group. Furthermore, the HOTAIR overexpression and control plasmids were constructed by GenePharma. The cells were transfected with the plasmids with 2.5 µl of Lipofectamine 2000 (Invitrogen, USA) and treated with 8 μg/ml of ginsenoside-Rg3, which was also added to the transfected cells. This experiment was performed in triplicate. After 6-h incubation in RPMI-1640 at 37°C with 5% CO_2_.

### qRT-PCR

qRT-PCR was employed to detect the mRNA expression of lncRNA-HOTAIR. According to the manufacturer’s protocol, RNA was isolated by RNeasy Mini Kit (Qiagen, Germany), and the RNA concentration was determined by spectrophotometer nanodrop 2000. The M-MLV was applied to synthesize cDNA through reverse transcription. The running order is 43°C, 30 min; 97°C, 5 min; and 5°C, 5 min. PrimeScript™ RT-PCR Kit (TaKaRa, Japan) was used for RT-PCR. The running order is 95°C, 5 min; 95°C, 30 s, 40 cycles; 59°C, 30 s; and 72°C, 30 s. Each experiment was performed in triplicate. The abundance of gene expression was determined by 2^−ΔΔCt^ relative quantification [19]. GAPDH was regarded as an internal control.

### Western blot

Western blot was applied according to previous study [20]. Cells were lysed by RIPA (Thermo Fisher Scientific, USA). Total protein levels were verified by BCA kit (Thermo Fisher Scientific, USA). 30 µg of proteins was electrophoresed in 15% SDS–PAGE and then transferred to polyvinylidene difluoride membranes (Millipore, Netherlands). The membrane was blocked with 5% skimmed milk for 2 h. Subsequently, the membrane was incubated with the primary antibodies including anti-MMP2 (ab92536, 1:1000, Abcam, USA), -MMP9 (ab38898, 1:1000, Abcam, USA), AKT (ab8805, 1:10,000, Abcam, USA), -PI3K (ab32089, 1:1000, Abcam, USA), -p-AKT (phospho S474, 1:1000, Abcam, USA), and -p-PI3K (phospho Y607, 1:1000, Abcam, USA) for 1 h at indoor temperature. Furthermore, the membrane was incubated with secondary antibody (ab6721, 1:2000, Abcam, USA) conjugated with HRP for 45 min at indoor temperature. Then, ECL Western blotting kit (Santa Cruz Biotechnology, Inc.) was adopted for membrane stain and analyzed with ImageJ software. Each experiment was repeated three times.

### MTT

We measured cell proliferation by MTT assay as described by Kumar et al. [21]. The transfected SMMC-7721 and SK-Hep-1 cells were placed into 24-well plates and incubated at 37°C with 5% CO_2_ for 48 h. Cells were treated with 1, 2, 4, 8, and 16 μg/ml of ginsenoside-Rg3. Subsequently, MTT Cell Viability Assay Kit (Thermo Fisher Scientific, USA) was performed according to the manufacturer's protocol. Moreover, we detected OD value under 570 nm wavelength using a microplate reader at the 0, 12, 24, 48 and 72 h time points.

Then, the lowest cell viability was in the group treated with 8 μg/ml ginsenoside-Rg3; therefore, 8 μg/ml ginsenoside-Rg3 was selected for the following experiments.

### Scratch assay & transwell assay

We evaluated the migration and invasion ability of SMMC-7721 and SK-Hep-1 cells through Scratch assay and transwell assay according to Ni et al. [22]. The SMMC-7721 and SK-Hep-1 cells were placed into 96-well plates and a monolayer cell culture was obtained. Then, a scratch across the center of the wells was generated by a new 1-ml pipette tip. Therefore, the width of the scratch was equal to the outer diameter of the tip. Then, the cells were incubated at 37°C with 5% CO_2_ for 72 h. The migrated cells were captured with a microscope (Olympus, Tokyo, Japan).

Furthermore, the invasion ability of SMMC-7721 cells and SK-Hep-1 cells was measured by transwell assay. The transwell cell culture inserts (8-mm pore size; Falcon; BD Biosciences) were seeded into the wells of 96-well plates to create the separate upper and lower chambers. Furthermore, the upper side of the membrane was pre-coated with Matrigel (BD Biosciences) and incubated for 1 h at 37°C for gel formation. The membrane was hydrated with FBS 2 h. Then, RPMI-1640 (600 µl) containing 10% FBS and 1 × 10^5^ cells/well was added to the lower and upper chambers, respectively. Then, we calculated invading cells by counting chamber after 48 h incubation.

### Statistical methods

All experimental data were presented as the mean ± standard deviation (SD) and analyzed by using GraphPad Prism version 5.01 (GraphPad Software, La Jolla, CA, USA). Moreover, the data comparison was employed by t-test and one-way analysis. *p* < 0.05 was taken as being statistically significant difference.

## Results

The present study aimed to explore the effect of ginsenoside-Rg3 on the tumorigenic behavior of the HCC cells. We hypothesized that ginsenoside-Rg3 downregulated the lncRNA HOTAIR expression and inhibited the proliferation, migration and invasion of the SMMC-7721 and SK-Hep-1 cells via regulating the PI3k/AKT signaling pathway.

### Cell viability under different concentrations of ginsenoside-Rg3

**Figure 1.**
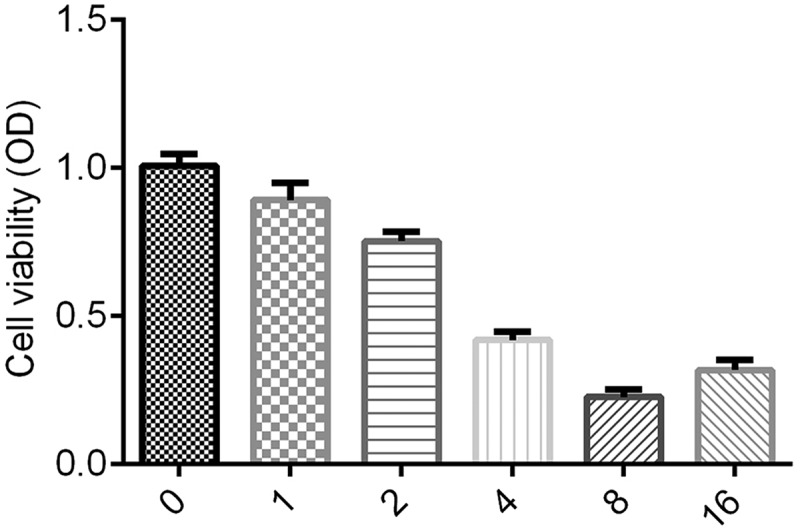
Cell viability under different concentrations of ginsenoside Rg3

SMMC-7721 Cells were treated with 1, 2, 4, 8 and 16 μg/ml of ginsenoside-Rg3. As shown in Figure 1, in inhibiting the cell viability of SMMC-7721, 8 μg/ml of ginsenoside-Rg3 was more effective. Therefore, the 8 μg/ml of ginsenoside-Rg3 was selected for the following performance.

The SMMC-2271 cells were treated with 0, 1, 2, 4, 8 and 16 μg/ml ginsenoside Rg3 and the cell viability was evaluated by MTT assay after incubation.

### Reduced lncRNA-HOTAIR expression by ginsenoside-Rg3

**Figure 2.**
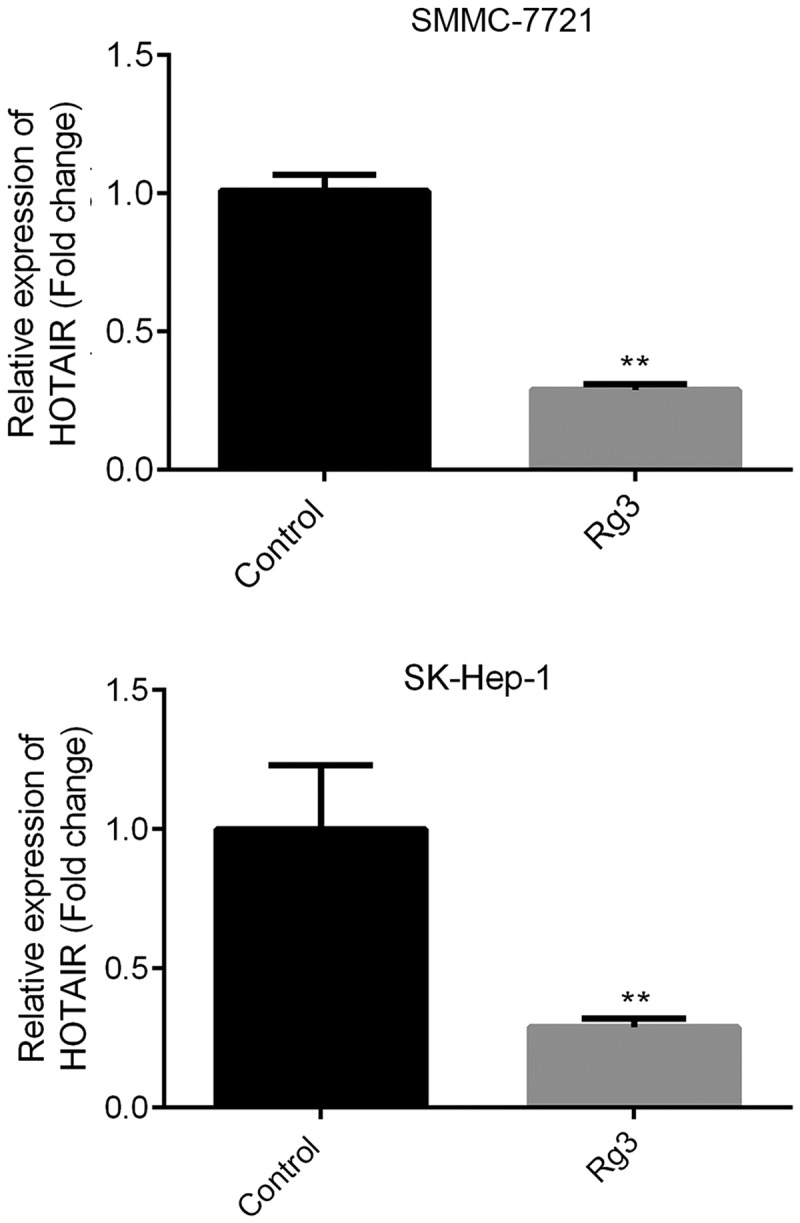
Reduced lncRNA-HOTAIR expression by ginsenoside Rg3

Using qRT-PCR, we evaluated the relative expression of lncRNA-HOTAI. Figure 2 indicates that ginsenoside-Rg3 could significantly suppress the expression of lncRNA-HOTAIR compared to the control group. Therefore, we might conclude that ginsenoside Rg3 could inhibit the lncRNA-HOTAIR expression in SMMC-7721 cells and SK-Hep-1 cells.

SMMC-7721 and SK-Hep-1 cells were incubated with 8 μg/ml ginsenoside Rg3 in RPMI-1640 (600 µl) containing 10% FBS at 37°C with 5% CO_2_. The relative expression of lncRNA-HOTAIR was evaluated by qRT-PCR. Control: non-treated group; ginsenoside Rg3: cells treated with 8 μg/ml ginsenoside Rg3. **p < 0.05 vs. control group.

### LncRNA-HOTAIR overexpression system

**Figure 3.**
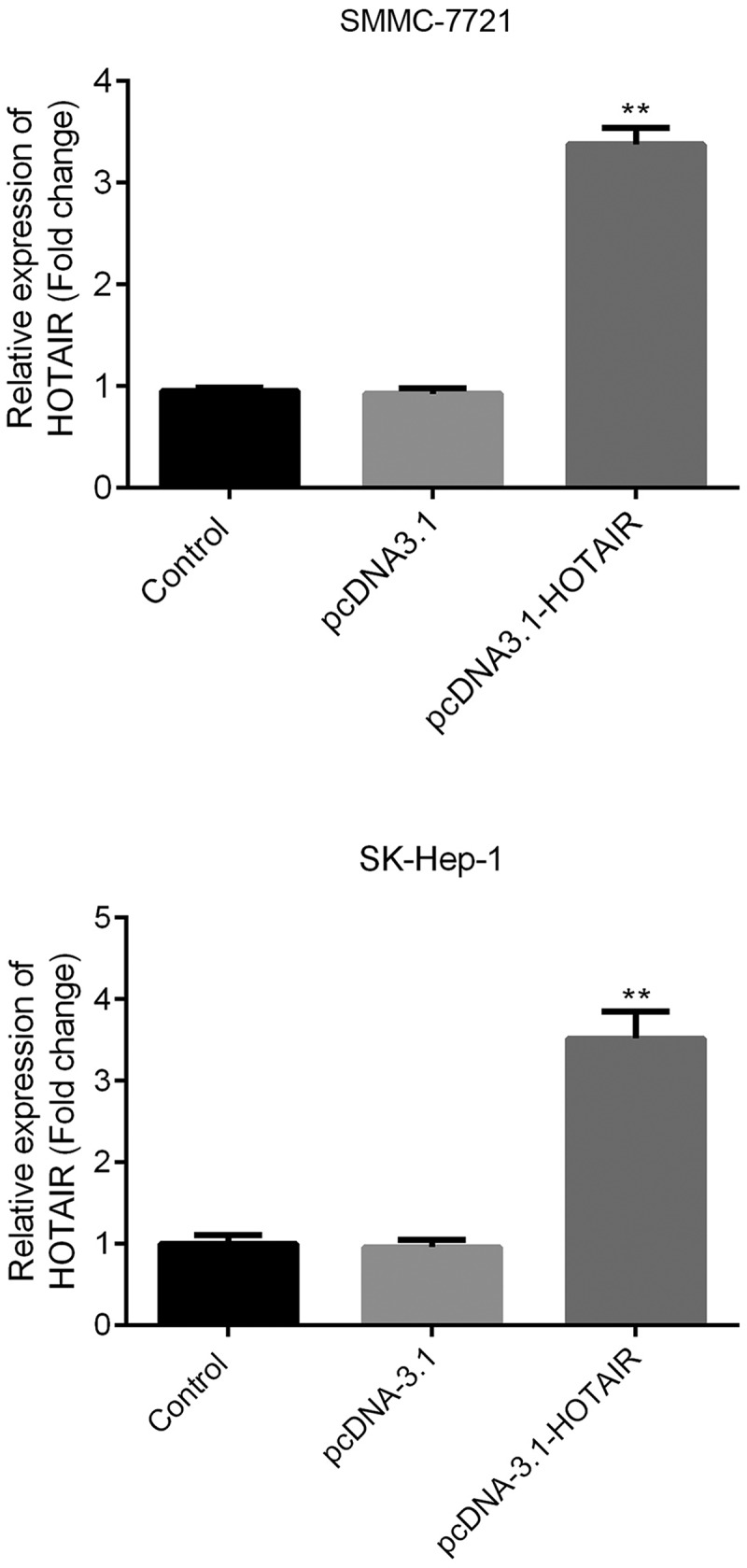
LncRNA-HOTAIR overexpression system

The lncRNA-HOTAIR-negative control plasmids and lncRNA-HOTAIR overexpression plasmids were added to SMMC-7721 cells and SK-Hep-1 cells. Figure 3 shows that the lncRNA-HOTAIR was dramatically overexpressed, which means that the lncRNA-HOTAIR overexpression plasmids were successfully constructed and can be applied to the following experiments.

The lncRNA-HOTAIR-negative and overexpression plasmids were added to SMMC-7721 cells and incubate with RPMI-1640 (600 µl) containing 10% FBS at 37°C with 5% CO2 for 48 h. The relative expression of lncRNA-HOTAIR was determined by qRT-PCR. Control: non-treated group; pcDNA3.1: cells treated with lncRNA-HOTAIR-negative control plasmids; pcDNA3.1-HOTAIR: cells treated with lncRNA-HOTAIR overexpression plasmids. **p < 0.05 vs. control group.

### Inhibited cell proliferation rate by ginsenoside Rg3

**Figure 4.**
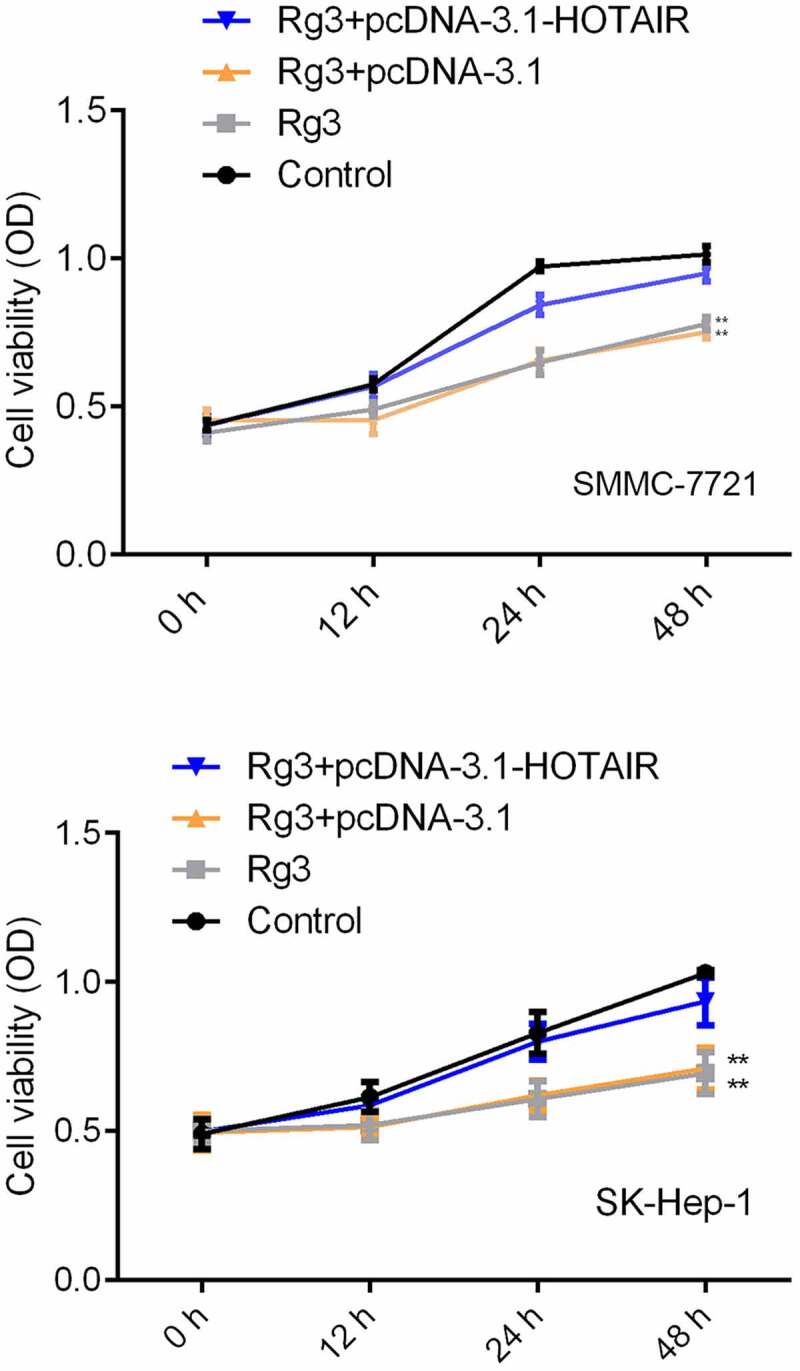
Inhibited cell proliferation rate by ginsenoside Rg3

The SMMC-7721 cells and SK-Hep-1 cells were treated with lncRNA-HOTAIR-negative control plasmids, ginsenoside Rg3 and lncRNA-HOTAIR overexpression plasmids and cultured RPMI-1640 (600 µl) containing 10% FBS for 48 h at 37°C/5% CO_2_. The viability of the cell was measured by MTT assay. Figure 4 demonstrates that the SMMC-7721 cell viability was significantly reduced in ginsenoside Rg3 groups and lncRNA-HOTAIR-negative control groups compared to the control group. Therefore, ginsenoside Rg3 could induce the decreased cell viability, which might inhibit the development of hepatoma carcinoma.

The cell viability was determined by MTT assay after 0, 12, 24 and 48 h incubation with RPMI-1640 (600 µl) containing 10% FBS at 37°C with 5% CO_2_. Control: non-treated group; Ginsenoside Rg3: cells treated with 8 μg/ml ginsenoside Rg3. Rg3+ pcDNA3.1: cells treated with 8 μg/ml ginsenoside Rg3 and lncRNA-HOTAIR-negative control plasmids; Rg3+ lncRNA-HOTAIR-negative control plasmids: 8 μg/ml ginsenoside Rg3 and lncRNA-HOTAIR overexpression plasmids.

### The migration and invasion ability of cell inhibited by ginsenoside Rg3

**Figure 5.**
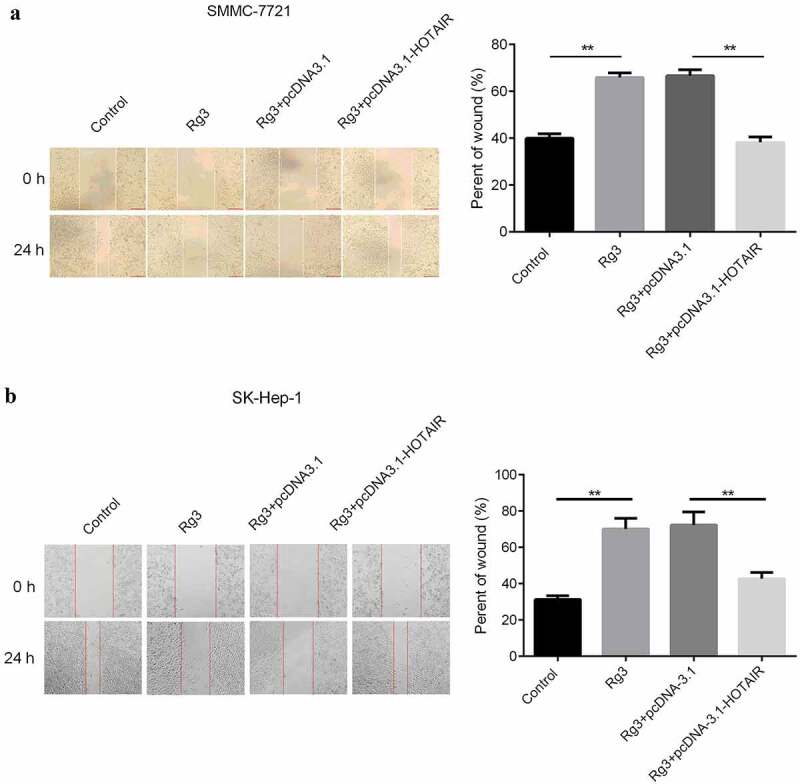
Inhibited cell migration by ginsenoside Rg3

**Figure 6.**
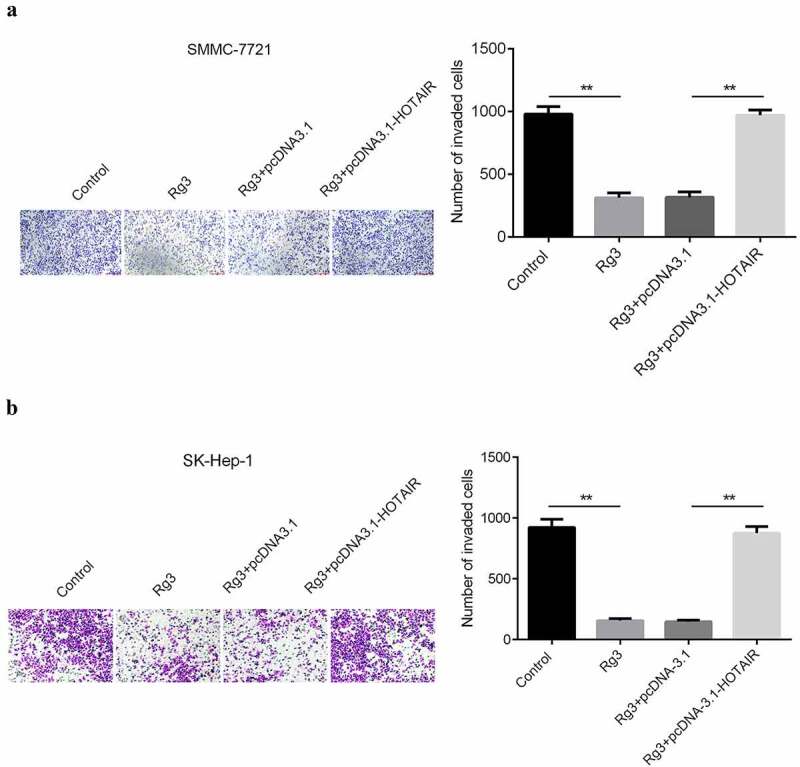
Inhibited cell invasion ability by ginsenoside Rg3

After incubation with lncRNA-HOTAIR-negative control plasmids, ginsenoside Rg3 and lncRNA-HOTAIR overexpression plasmids for 48 h, the migration and invasion ability of cells were evaluated with scratch and transwell assay. In Figures 5 and 6, the results indicated that a huge reduction in the migration and invasion ability of cells was found in ginsenoside Rg3 and lncRNA-HOTAIR-negative control groups compared with control and lncRNA-HOTAIR overexpression groups. Therefore, ginsenoside Rg3 might efficiently inhibit the migration and invasion of SMMC-7721 and SK-Hep-1 cells.

(a)–(b): The cell migration ability was determined by scratch assay after 48 incubation with RPMI-1640 (600 µl) containing 10% FBS at 37°C with 5% CO_2_. Control: non-treated group; Ginsenoside Rg3: cells treated with 8 μg/ml ginsenoside Rg3. Rg3+ pcDNA3.1: cells treated with 8 μg/ml ginsenoside Rg3 and lncRNA-HOTAIR-negative control plasmids; Rg3+ lncRNA-HOTAIR-negative control plasmids: 8 μg/ml ginsenoside Rg3 and lncRNA-HOTAIR overexpression plasmids.

(a)–(b): The invasion ability was determined by a transwell assay after 48 incubation with RPMI-1640 (600 µl) containing 10% FBS at 37°C with 5% CO_2_. Control: non-treated group; Ginsenoside Rg3: cells treated with 8 μg/ml ginsenoside Rg3. Rg3+ pcDNA3.1: cells treated with 8 μg/ml ginsenoside Rg3 and lncRNA-HOTAIR-negative control plasmids; Rg3+ lncRNA-HOTAIR-negative control plasmids: 8 μg/ml ginsenoside Rg3 and lncRNA-HOTAIR overexpression plasmids.

### Inhibited expression of MMP2, MMP9, p-AKT, and p-PI3K by ginsenoside Rg3

**Figure 7.**
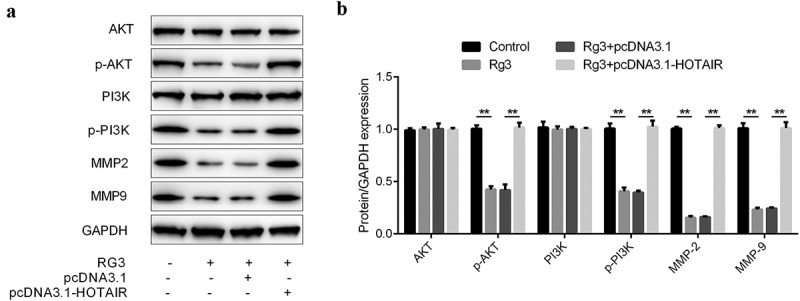
Inhibited expression of MMP2, MMP9, p-AKT, and p-PI3K by ginsenoside Rg3

The protein expression was determined by Western blot. There were four groups: control group, ginsenoside Rg3 group, ginsenoside Rg3 plus lncRNA-HOTAIR overexpression group and ginsenoside Rg3 plus lncRNA-HOTAIR-negative control group. In Figure 7, the dramatically inhibited expression of MMP2, MMP9, p-AKT, and p-PI3K was observed in ginsenoside Rg3 groups compared to the control group. Furthermore, the MMP2, MMP9, p-AKT, and p-PI3K were significantly increased in lncRNA-HOTAIR overexpression group treated with ginsenoside Rg3. Hence, ginsenoside Rg3 could induce the reduced expression of MMP2, MMP9, p-AKT, and p-PI3K in SMMC-7721 cells.

(a): The protein level of MMP2, MMP9, PI3K/AKT, p-AKT and p-PI3K was determined by Western blot. Expression of proteins by Western blot. (b): Quantitative analysis of (a). Control: non-treated group; Ginsenoside Rg3: cells treated with 8 μg/ml ginsenoside Rg3. Rg3+ pcDNA3.1: cells treated with 8 μg/ml ginsenoside Rg3 and lncRNA-HOTAIR-negative control plasmids; Rg3+ lncRNA-HOTAIR-negative control plasmids: 8 μg/ml ginsenoside Rg3 and lncRNA-HOTAIR overexpression plasmids. **p < 0.05 vs. control group. ## *p* < 0.05 vs. Rg3+ lncRNA-HOTAIR-negative control group.

## Discussion

In our study, we first found that ginsenoside Rg3 downregulates the lncRNA-HOTAIR expression and relieves the carcinogenic behaviors of HCC cells via inhibiting the PI3k/AKT signaling pathway. Our results demonstrated that ginsenoside Rg3 was a potent multi-target antitumor agent for the treatment of HCC in conclusion.

The indefinite proliferative ability of HCC cells is the main factor leading to the poor curative effect and recurrence of HCC. Effective inhibition of cell growth and metastasis is the main strategy to alleviate the occurrence and development of HCC [23]. In recent years, a growing number of studies have reported that natural extracts have excellent therapeutic effect on HCC. For example, chrysin can prevent sphere formation in SMMC-7721 cells [24]. Berbamine promoted the apoptosis of SMMC-7721 cell via mitochondrial signaling pathway [25]. Xu et al. [26] also suggested that isovitexin induces apoptosis and suppresses the proliferation of SK-Hep-1 cells. In addition, studies have shown that ginsenosides exhibit significant antitumor effect. Dai et al. [27] found that ginsenoside Rb2 suppresses epithelial–mesenchymal transition development of colorectal cancer cells. Similarly, Li et al. [28] demonstrated that ginsenoside Rh2 can transform tumor-associated macrophages from M2 to M1 subgroup and inhibit the migration ability of lung cancer cells. In this study, we found that ginsenoside Rg3 prevents the growth and metastasis of the HCC cells, which was also found in the study by Shan et al. [29].

As a prognostic circulating marker and potential therapeutic target in patients with tumor diseases, LncRNA has been studied currently in various cancers [30]. Han et al. [31] confirmed that LncRNA-DNAJC3-AS1 promotes the progression and development of colon cancer. Zhang et al. suggested that LncRNA H19 is considered to be closely related to the extent of malignant degree [32]. These reports indicated that LncRNA may be an effective target for tumor molecular therapy. Previously, it was studied that lncRNA HOTAIR was associated with metastasis, differentiation, and early recurrence. Furthermore, knockdown of HOTAIR lncRNA diminishes cell proliferation and is associated with decreased levels of MMP-9 and vascular endothelial growth factor protein, which are crucial for cell motility and metastasis [33]. According to its asymmetric carbon atom C20, Ginsenoside Rg3 can be divided into the R type and S type. And due to the activation or inhibition of different gene expression, Ginsenoside Rg3 also has diverse pharmacological effects in vivo and in vitro [34]. According to our report, through decreasing the expression of epidermal growth and upregulating the protein expression of pro-apoptotic P53, ginsenoside Rg3 can inhibit cancer cell proliferation and induce apoptosis in HCC [34]. In the present study, we verified that 8 μg/ml ginsenoside Rg3 could induce the downregulation of lncRNA-HOTAIR, leading to the reduced rate of cell proliferation, migration and invasion via PI3k/AKT signaling pathway.

However, due to the limited time and resources, our experimental design has some shortcomings. For instance, ginsenoside Rg3 has various targets in cancers, and lots of targets may result in the same or opposite effects on cancer. In our present study, the group of lncRNA-HOTAIR knockdown lacked. Therefore, we cannot compare the cell proliferation, invasion and migration ability between ginsenoside Rg3 group and ncRNA-HOTAIR knockdown group. Thus, we could not identify the exact effects of ginsenoside Rg3 on SMMC-7721 cells and SK-Hep-1 cells and the associated impacts on HCC.

## Conclusion

To sum up, we found that ginsenoside Rg3 significantly inhibits the proliferation of the SMMC-7721 and SK-Hep-1 cells. 8 μg/ml ginsenoside Rg3 downregulated the lncRNA-HOTAIR expression and suppressed the growth and metastasis of the SMMC-7721 and SK-Hep-1 cells, while overexpression of lncRNA-HOTAIR reversed the role of ginsenoside Rg3. In addition, this study demonstrated that ginsenoside Rg3 relieves the carcinogenic behaviors of the SMMC-7721 and SK-Hep-1 cells by inhibiting the PI3k/AKT signaling pathway. Our study laid a theoretical basis for the further promotion and application of ginsenoside Rg3 and provided a novel insight for the research on the therapy of HCC.

## Highlights

1. Ginsenoside-Rg3 could significantly suppress the expression of lncRNA-HOTAIR
2. Ginsenoside Rg3 could induce the decreased cell viability
3. Inhibition of PI3k/AKT signaling pathway

## References

[1] Tsuchiya N, Sawada Y, Endo I, et al. Biomarkers for the early diagnosis of hepatocellular carcinoma. World J Gastroenterol. 2015;21(37):10573–10583. 26457017 10.3748/wjg.v21.i37.10573 PMC4588079

[2] Fujiwara N, Friedman SL, Goossens N, et al. Risk factors and prevention of hepatocellular carcinoma in the era of precision medicine. J Hepatol. 2018;68:526–549. 28989095 10.1016/j.jhep.2017.09.016 PMC5818315

[3] Wang N, Wang S, Li MY, et al. Cancer stem cells in hepatocellular carcinoma: an overview and promising therapeutic strategies. Ther Adv Med Oncol. 2018;10:1758835918816287. 30622654 10.1177/1758835918816287 PMC6304707

[4] Bertot LC, Adams LA. Trends in hepatocellular carcinoma due to non-alcoholic fatty liver disease. Expert Rev Gastroenterol Hepatol. 2019;13(2):179–187. 30791782 10.1080/17474124.2019.1549989

[5] De Matteis S, Ragusa A, Marisi G, et al. Aberrant metabolism in hepatocellular carcinoma provides diagnostic and therapeutic opportunities. Oxid Med Cell Longev. 2018;2018:7512159. 30524660 10.1155/2018/7512159 PMC6247426

[6] Hajjari M, Rahnama S. Association between SNPs of long non-coding RNA HOTAIR and risk of different cancers. Front Genet. 2019;10:113. 30873206 10.3389/fgene.2019.00113 PMC6403183

[7] Liu K, Zhao D, Wang D. LINC00528 regulates myocardial infarction by targeting the miR-143-3p/COX-2 axis. Bioengineered. 2020;11(1):11–18. 31833800 10.1080/21655979.2019.1704535 PMC6961595

[8] Ding J, Cao J, Chen Z, et al. The role of long intergenic noncoding RNA 00511 in malignant tumors: a meta-analysis, database validation and review. Bioengineered. 2020;11(1):812–823. 32713253 10.1080/21655979.2020.1795384 PMC8291795

[9] Hong Q, Li O, Zheng W, et al. LncRNA HOTAIR regulates HIF-1α/AXL signaling through inhibition of miR-217 in renal cell carcinoma. Cell Death Dis. 2017;8(5):e2772. 28492542 10.1038/cddis.2017.181 PMC5520706

[10] Bao X, Ren T, Huang Y, et al. Long chain non-coding RNA (lncRNA) HOTAIR knockdown increase miR-454-3p to suppress gastric cancer growth by targeting STAT3/Cyclin D1. Med Sci Monit. 2019;25:1537–1548. 30810117 10.12659/MSM.913087 PMC6402277

[11] Cheng D, Deng J, Zhang B, et al. LncRNA HOTAIR epigenetically suppresses miR-122 expression in hepatocellular carcinoma via DNA methylation. EBio-Medicine. 2018;36:159–170. 10.1016/j.ebiom.2018.08.055 PMC6197532 30195653

[12] Zhong DN, Luo YH, Mo WJ, et al. High expression of long non-coding HOTAIR correlated with hepatocarcinogenesis and metastasis. Mol Med Rep. 2018;17(1):1148–1156. 29115524 10.3892/mmr.2017.7999

[13] Yang Z, Zhou L, Wu LM, et al. Overexpression of long non-coding RNA HOTAIR predicts tumor recurrence in hepatocellular carcinoma patients following liver transplantation. Ann Surg Oncol. 2011;18(5):1243–1250. 21327457 10.1245/s10434-011-1581-y

[14] Cao Y, Ye Q, Zhuang M, et al. Ginsenoside Rg3 inhibits angiogenesis in a rat model of endometriosis through the VEGFR-2-mediated PI3K/Akt/mTOR signaling pathway. PLoS One. 2017;12(11):e0186520. 29140979 10.1371/journal.pone.0186520 PMC5687597

[15] Sun MY, Song YN, Zhang M, et al. Ginsenoside Rg3 inhibits the migration and invasion of liver cancer cells by increasing the protein expression of ARHGAP9. Oncol Lett. 2018;17:965–973. 30655855 10.3892/ol.2018.9701 PMC6313058

[16] Dai Y, Wang W, Sun Q, et al. Ginsenoside Rg3 promotes the antitumor activity of gefitinib in lung cancer cell lines. Exp Ther Med. 2018;17(1):953–959. 30651886 10.3892/etm.2018.7001 PMC6307378

[17] Xu W, Ni Z, Zhang M, et al. The role of polymorphisms in genes of PI3K/Akt signaling pathway on prostate. J Cancer. 2019;10(4):1023–1031. 30854108 10.7150/jca.26472 PMC6400800

[18] Sun XF, Shao YB, Liu MG, et al. High-concentration glucose enhances invasion in invasive ductal breast carcinoma by promoting Glut1/MMP2/MMP9 axis expression. Oncol Lett. 2017;13(5):2989–2995. 28521406 10.3892/ol.2017.5843 PMC5431328

[19] Livak KJ, Schmittgen TD. Analysis of relative gene expression data using real-time quantitative PCR and the 2(-Delta Delta C(T)) method. Methods. 2001;25(4):402–408. 11846609 10.1006/meth.2001.1262

[20] Taylor SC, Posch A. The design of a quantitative western blot experiment. Biomed Res Int. 2014;2014:361590. 24738055 10.1155/2014/361590 PMC3971489

[21] Kumar P, Nagarajan A, Uchil PD. Analysis of cell viability by the MTT assay. Cold Spring Harb Protoc. 2018;2018(6). DOI:10.1101/pdb.prot095505 29858338

[22] Ni X, Ding Y, Yuan H, et al. Long non-coding RNA ZEB1-AS1 promotes colon adenocarcinoma malignant progression via miR-455-3p/PAK2 axis. Cell Prolif. 2020;53(1):e12723. 31828845 10.1111/cpr.12723 PMC6985675

[23] Kim DW, Talati C, Kim R. Hepatocellular carcinoma (HCC): beyond sorafenib-chemotherapy. J Gastrointest Oncol. 2017;8(2):256–265. 28480065 10.21037/jgo.2016.09.07 PMC5401857

[24] Zhang Y, Chen F, Xiao X, et al. Chrysin inhibits sphere formation in SMMC-7721 cells via modulation of SHP-1/STAT3 signaling pathway. Cancer Manag Res. 2019;11:2977–2985. 31114345 10.2147/CMAR.S193647 PMC6497861

[25] Cao Y, Cao J, Yu B, et al. Berbamine induces SMMC-7721 cell apoptosis via upregulating p53, downregulating survivin expression and activating mitochondria signaling pathway. Exp Ther Med. 2018;15(2):1894–1901. 29434780 10.3892/etm.2017.5637 PMC5776608

[26] Xu C, Cao X, Cao X, et al. Isovitexin inhibits stemness and induces apoptosis in hepatocellular carcinoma SK-Hep-1 spheroids by upregulating miR-34a expression. Anticancer Agents Med Chem. 2020;20(14):1654–1663. 32329692 10.2174/1871520620666200424123139

[27] Dai G, Sun B, Gong T, et al. Ginsenoside Rb2 inhibits epithelial-mesenchymal transition of colorectal cancer cells by suppressing TGF-β/Smad signaling. Phytomedicine. 2019;56:126–135. 30668333 10.1016/j.phymed.2018.10.025

[28] Li H, Huang N, Zhu W, et al. Modulation the crosstalk between tumor-associated macrophages and non-small cell lung cancer to inhibit tumor migration and invasion by ginsenoside Rh2. BMC Cancer. 2018;18(1):579. 29783929 10.1186/s12885-018-4299-4 PMC5963019

[29] Shan K, Wang Y, Hua H, et al. Ginsenoside Rg3 combined with oxaliplatin inhibits the proliferation and promotes apoptosis of hepatocellular carcinoma cells via downregulating PCNA and cyclin D1. Biol Pharm Bull. 2019;42(6):900–905. 30930425 10.1248/bpb.b18-00852

[30] Zhang C, Ren X, Zhang W, et al. Prognostic and clinical significance of long non-coding RNA SNHG12 expression in various cancers. Bioengineered. 2020;11(1):1112–1123. 33124951 10.1080/21655979.2020.1831361 PMC8291808

[31] Han B, Ge Y, Cui J, et al. Down-regulation of lncRNA DNAJC3-AS1 inhibits colon cancer via regulating miR-214-3p/LIVIN axis. Bioengineered. 2020;11(1):524–535. 32352854 10.1080/21655979.2020.1757224 PMC7202691

[32] Zhang H, Yu Y, Zhang K, et al. Targeted inhibition of long non-coding RNA H19 blocks anaplastic thyroid carcinoma growth and metastasis. Bioengineered. 2019;10(1):306–315. 31299871 10.1080/21655979.2019.1642722 PMC6650201

[33] Abbastabar M, Sarfi M, Golestani A, et al. lncRNA involvement in hepatocellular carcinoma metastasis and prognosis. EXCLI J. 2018;17:900–913. 30564069 10.17179/excli2018-1541 PMC6295623

[34] Teng S, Wang Y, Li P, et al. Effects of R type and S type ginsenoside Rg3 on DNA methylation in human hepatocarcinoma cells. Mol Med Rep. 2017;15(4):2029–2038. 28260016 10.3892/mmr.2017.6255 PMC5364960

